# From Tobacco Cigarettes to Electronic Cigarettes: The Two Sides of a Nicotine Coin

**DOI:** 10.3389/froh.2021.790634

**Published:** 2021-11-26

**Authors:** Ahmad Besaratinia

**Affiliations:** Department of Population and Public Health Sciences, USC Keck School of Medicine of the University of Southern California, Los Angeles, CA, United States

**Keywords:** electronic cigarette, epidemic, nicotine, public health, tobacco, youth vaping

## Abstract

Tobacco smoking-related diseases, including cardiovascular disease, pulmonary disease, stroke, and cancer in multiple organ sites, are the leading causes of preventable death, worldwide. Youth electronic cigarette use (vaping) is an evolving public health problem in the United States and around the world. Many of the same toxicants and carcinogens present in tobacco smoke are also found in electronic cigarette vapor, although mostly at substantially lower levels. The reduced concentrations of these chemicals in electronic cigarette vapor may imply lower health risk; however, they cannot equate to no risk. To date, the long-term health consequences of vaping are largely unknown. This “*Perspective”* provides a concise chronology of events leading up to an unprecedented global challenge, namely the convergence of global tobacco epidemic and youth vaping epidemic. Current state of knowledge, outstanding questions in the field, present challenges, and future directions in research are highlighted. The existing data show a continued and dynamic evolution of the converged epidemics. The goal should be to prevent youth vaping while improving smoking cessation strategies. In smokers who are unable or unwilling to quit smoking, the objective should be to provide “*provably”* safe or less-harmful alternatives, which should “completely” or “substantially” substitute tobacco cigarettes.

## A Historical Introduction

Tobacco industry has continuously used product modifications, such as manipulating smoke pH with various chemical additives, to make combustible cigarettes more appealing to novice users [1, 2]. For decades, Philip Morris and other tobacco companies have used ammonia as a relatively innocuous additive for a variety of purposes, including augmenting certain flavors, cutting costs by expanding or “puffing up” the volume of cured tobacco leaves, preparing reconstituted tobacco sheet (“recon”), denicotinizing tobacco (i.e., to reduce the amount of nicotine in tobacco leaf), and even lowering/removing tobacco smoke carcinogens [2]. In the early 1960s, while evaluating the impact of ammoniated recons used in Marlboro cigarettes, Philip Morris discovered that freebase or unbound nicotine, as opposed to nicotine bound to other molecules (e.g., salt), is more volatile and vaporizable, thus being highly bioavailable [2, 3]. This led to the development of low-yield cigarettes (with reduced tar and nicotine content) that still had the nicotine kick necessary to keep customers “satisfied” (i.e., addicted) [2]. The freebased low-yield version of Marlboro cigarettes became the world's most popular cigarette; Marlboro has remained the top selling cigarette brand in the world since 1972 [4]. The commercial success of Marlboro persuaded the rest of the tobacco industry to utilize ammonia to convert nicotine to its freebase form, as part of a new process for manufacturing cigarettes. However, this was achieved only after competitors uncovered Philip Morris' “*secret”* of freebasing nicotine in cigarettes by reverse engineering the chemistry of Marlboro cigarettes [2].

More than half a century later, a relatively unknown vape company, Juul Labs Inc., recognized the utility of salt-based (protonated) nicotine for a novel electronic cigarette (e-cig) device, called JUUL [5]. Started in 2017, Juul Inc. is a spin off company from Pax Labs, which was a manufacturer of vaporizing devices for cannabis and loose-leaf tobacco; Pax Labs was preceded by Ploom as the original company for e-cig development [5]. Remarkably, JUUL's use of salt-based nicotine, which is significantly less aversive than freebase nicotine, made it very quickly popular among naïve users, particularly adolescents and youth [6]. The high content of nicotine in JUUL, which was claimed to be equivalent to nicotine content of a pack of 20 cigarettes, also made JUUL highly appealing to adult smokers, seeking a putatively less-harmful alternative to combustible cigarettes [6]. Shortly after its launch, JUUL became the preeminent vaping product on the market and a dominant player in the vaping industry [5, 6]. In December 2018, Altria, one of the world's largest cigarette manufacturers and the parent company of Philip Morris USA, acquired a 35% stake in JUUL [7]. This made Altria a major force in both the tobacco and vaping markets [7].

Nearly 1 year later, the US Surgeon General declared “youth vaping” an “*epidemic”* in the United States [8], and the US Food and Drug Administration (FDA) called JUUL a particular cause for concern [9]. Not long after, in August 2019, the Centers for Disease Control and Prevention (CDC), the FDA, state and local health departments, and other clinical and public health partners reported a nation-wide outbreak of vaping-related severe lung illnesses and deaths, also referred to as “e-cig, or vaping, product use-associated lung injury (EVALI)” [10]. Most EVALI cases reported using e-cig products containing vitamin E acetate and tetrahydrocannabinol (THC), the principal psychoactive component of cannabis. However, the CDC did not rule out the etiologic involvement of other substances present in non-THC containing e-cig products [10]. The EVALI outbreak lasted for several months, but declined considerably by February 2020 [10].

## Convergence of Two Epidemics

The above timelines capture the evolution of events leading up to the convergence of global tobacco epidemic and youth vaping epidemic in the United States. Philip Morris' pioneering of free-basing nicotine technology and manufacturing the best-selling Marlboro cigarettes [2] followed by JUUL's becoming the leader of vaping industry and subsequent acquisition of its stake (35%) by Altria [7] made the giant of the tobacco industry a focus of attention for two converging epidemics. Whereas, the global tobacco epidemic kills nearly eight million people, annually [11], youth vaping epidemic is a major public health concern in the United States and many parts of the world [12].

## Litigations Concerning Altria's Ownership of Combustible and Electronic Cigarettes

Having the sole or major ownership of products responsible for both the global tobacco epidemic and youth vaping epidemic, Altria is facing various litigations [6]. On April 1, 2020, the Federal Trade Commission (FTC) filed an administrative complaint alleging that Altria Group Inc. and Juul Labs, Inc. entered a series of agreements, including a merger that turned Altria into JUUL's largest shareholder [13]. The FTC alleged that Altria's acquisition of JUUL shares and associated agreements together constitute an unreasonable restraint of trade in violation of Section 1 of the Sherman Act and Section 5 of the FTC Act, and substantially lessen competition in violation of Section Vaping Regulations of the Clayton Act [7, 13]. The allegations will be tried in a formal hearing before an administrative law judge. The administrative trial is scheduled to begin in 2021 [13].

## Prevalence of Smoking and Youth Vaping

Worldwide, there are around 1.1 billion current cigarette smokers aged 15 or older, of whom 942 million are males and 175 million are females [11]. In 2019, there were an estimated 155 million smokers aged between 15 and 24 years – equivalent to 20.1% of young men and 5.0% of young women, globally. Two-thirds (65.5%) of all current smokers began smoking by age 20, and 89% of smokers began by age 25, highlighting a critical age window during which individuals develop nicotine addiction and transition to become established smokers [14–16].

Globally, 7.4 trillion cigarette-equivalents of tobacco (combining smoked tobacco products, including manufactured cigarettes, hand-rolled cigarettes, cigars, cigarillos, pipes, shisha, and regional products, such as bidis and kreteks) were consumed in 2019, amounting to 20.3 billion each day. Countries with the highest consumption per person were mostly in Europe. One in three male and one in five female smokers consumed 20 or more cigarette-equivalents per day, worldwide. The 10 countries with the largest number of tobacco smokers, which together comprised nearly two-thirds of the global tobacco smoking population in 2019, were China, India, Indonesia, the United States, Russia, Bangladesh, Japan, Turkey, Vietnam, and the Philippines—one in three current tobacco smokers (341 million) lived in China. In many countries, progress in reducing the prevalence of smoking did not keep a pace with population growth, resulting in significant rises in the number of young smokers. India, Egypt, and Indonesia had the largest absolute increases in number of young male smokers. Turkey, Jordan, and Zambia had the largest increases in number of young female smokers. Over half of countries, worldwide, showed no progress in reducing smoking among 15–24 years old [14–16].

Youth vaping is an evolving public health problem in the United States and around the world [12]. Results from the 2020 National Youth Tobacco Survey (NYTS) [17] and Monitoring the Future (MTF) survey [18] showed that nearly 3.6 million American teens were current (past 30 days) users of e-cigs, of whom 80% reported using flavored products, such as fruit, mint, menthol, and candy, desserts, or other sweet-flavored e-liquids [17]. Specifically, one in five U.S. high school students and one in ten middle school students reported current use of e-cigs in 2020 [17, 18]. The CDC and FDA analysis of the 2021 NYTS, conducted during January 18 – May 21, 2021, showed an estimated 2.06 million U.S. middle and high school students reporting current use of e-cigs [19]. The authors, however, cautioned that because the 2021 NYTS was fully conducted amid the COVID-19 pandemic through mostly online data collection, estimates from this year's survey should not be compared to previous NYTS survey waves that were primarily conducted on school campuses [19].

Following a same trend, the percentage of college-age youth who vape nicotine, has risen dramatically in recent years. Between 2017 and 2019, the 30-day prevalence of e-cig use increased from 6 to 22% among college students, and from 8 to 18% among 19 to 22 year-olds not in college [20]. Together, these data indicate a continued and dynamic evolution of the global tobacco epidemic and youth vaping epidemic [21, 22].

## Health Consequences: Smoking vs. Vaping

Tobacco smoking-related diseases, including cardiovascular disease, pulmonary disease, stroke, and cancer in multiple organ sites, such as the lung, mouth (oral cavity), throat, nose and sinuses, esophagus, bladder, kidney, and ureter, pancreas, stomach, liver, cervix and ovary, the bowel (colorectal cancer), and white blood cells (acute myeloid leukemia), are the leading causes of preventable death, worldwide [11]. In 2019, smoking was associated with 1.7 million deaths from ischemic heart disease, 1.6 million deaths from chronic obstructive pulmonary disease, 1.3 million deaths from tracheal, bronchus, and lung cancer, and nearly 1 million deaths from stroke. Approximately 87% of deaths attributable to tobacco smoking occurred among current smokers. Only 6% of global deaths attributable to smoking occurred among individuals who had quit smoking for at least 15 years [14–16]. This underscores the significant health benefits of smoking cessation, especially when achieved earlier in life and soon after the initiation of smoking.

Chemical analyses of e-cig vapor have revealed the presence of some of the same toxicants and carcinogens as those found in cigarette smoke, including carbonyl compounds, volatile organic compounds, free radicals, and heavy metals, albeit mostly at substantially lower concentrations [23, 24]. E-cig vapor also contains chemicals that are not found in cigarette smoke [25, 26]. The latter likely arise from the mixing and heating of humectants [e.g., propylene glycol and glycerol (vegetable glycerin); PG/VG] and flavorings present in e-liquid [27]. Overall, the reduced levels of toxicants and carcinogens in e-cig vapor are consistent with the fact that e-cigs, unlike traditional cigarettes, do not “burn” tobacco to produce inhalable materials [12]. This has led to the perception that e-cig use/vaping is safe or less-harmful than tobacco smoking [28, 29]. Whilst the lower levels of toxicants and carcinogens in e-cig vapor may imply mitigated health risk, they cannot, however, equate to no risk [6]. In fact, exposure to many of the same constituents of e-cig vapor, at various concentrations, has been associated with a wide range of cardiovascular-, immune-related (inflammatory), and respiratory diseases, and cancer [22, 30–34].

Emerging data indicate that e-cig use may adversely affect the pulmonary and cardiovascular systems [24, 35–39]. Also, limited evidence exists on the potential of e-cigs to cause carcinogenic effects [32, 33, 40–45]. To date, clinical studies have primarily investigated the acute effects of vaping [24, 34]. By design, epidemiologic studies have explored the “*association”* of e-cig use with disease outcomes [34, 37]. So, the “*causal”* relationship between vaping and disease pathology remains largely unknown [24, 46].

Currently, determining the long-term health consequences of vaping is a top research priority [24, 47]. Of utmost importance is the long-term effects of vaping, especially among adolescents whose developing brains are more susceptible to influences on learning, memory, and attention [48–52]. Future well-defined controlled experimental studies are needed to establish the mechanisms by which chronic vaping may lead to adverse health consequences in humans [53]. These investigations are expected to identify the constituent(s) of e-cig vapor, which are of most relevance to disease development [12].

## Risk-Benefit Analysis of Vaping vs. Smoking

Though it is generally accepted that e-cig use exposes the users to fewer and lower levels of toxicants and carcinogens as compared to smoking [24, 34], the net public health effect of vaping continues to be debated [46]. The scientific community, regulatory authorities, and the general public are faced with competing views on the health risks or benefits of vaping [12]. On the one hand, proponents of e-cigs advocate for: (I) “harm reduction” potential of vaping, especially for smokers who are unable/unwilling to quit; and (II) utility of vaping for smoking cessation [28, 29]. On the other hand, opponents argue against the adoption of an alternative tobacco product whose long-term health effects are yet to be determined [24, 34, 37, 54, 55]. To reach a consensus, more conclusive scientific evidence will be needed; the available data favor the stance that vaping is likely to be less harmful than smoking, with the caveat that it may still pose a health risk on users, which would otherwise be eliminated if neither product were used [6].

## Vaping Regulations

On June 22, 2009, with broad bipartisan support from Congress, President Obama signed into law the Family Smoking Prevention and Tobacco Control Act (FSPTCA), also known as “Tobacco Control Act” [56]. The FSPTCA granted FDA immediate authority and unprecedented power to regulate cigarettes, cigarette tobacco, roll-your-own tobacco, smokeless tobacco, and any other tobacco products that the agency, by regulation, deems to be subject to the law (2009). On August 8, 2016, when FDA's “Deeming Rule” took effect [57], many of the regulatory and statutory requirements that had been in place for manufacturers of the originally regulated tobacco products since passage of the FSPTCA in 2009, became applicable to the deemed products, including e-cigs and all other electronic nicotine delivery systems (ENDS), cigars, pipe tobacco, nicotine gels, hookah tobacco, and any future products meeting the statutory definition of “tobacco product.” The applicable statutory provisions include the requirement that deemed products that meet the definition of a new tobacco product must receive premarket authorization from the FDA to be legally marketed. The premarket authorization requires a Premarket Tobacco Product Application (PMTA) for any new tobacco product seeking an FDA marketing order [58].

In the deeming rule and subsequent guidance documents, FDA stated that it intended to defer enforcing the premarket review requirements, for a period of time, with respect to “deemed” new tobacco products that were on the market as of the effective date of the deeming rule [57]. This policy did not extend to deemed new tobacco products that entered the market after the rule's effective date (2016). Under a federal court order, manufacturers of deemed new tobacco products that were on the market as of the deeming rule's effective date, were required to submit premarket review applications by September 9, 2020 [59]. Following the court order, FDA accelerated its planning and preparation to receive a large number of applications by the premarket application deadline [60]. Accordingly, the agency received thousands of submissions, representing more than 6.5 million products by the set deadline. Per the court order, products for which applications were submitted by the deadline of September 9, 2020, could generally remain on the market for up to a year from the date of the application—or until September 9, 2021, at the latest—pending FDA review, although FDA retains enforcement discretion [59].

Over the last year, FDA has worked to review thousands of PMTAs, representing products from roughly 500 companies [61]. The vast majority of the PMTAs are for ENDS products. Under the PMTA pathway, manufacturers or importers must demonstrate to the FDA that permitting the marketing of the new tobacco product(s) would be “appropriate for the protection of the public health,” among other things [58]. That statutory standard requires the FDA to consider the risks and benefits to the population as a whole, including users and non-users of tobacco products. The FDA's review of PMTAs includes assessment of a tobacco product's components, ingredients, additives, toxicological profile, and health effects, as well as its manufacturing, packaging, and labeling processes, and data from consumer perception research (if available), and the applicant's description of marketing plans for the product [58]. When reviewing PMTAs for ENDS products, FDA's task is to follow the direction Congress provided in the law by taking into account the increased or decreased likelihood that existing users of tobacco products will stop using such products, as well as the increased or decreased likelihood that those who do not use tobacco products will start using such products. In making this determination, the impact of such products on youth initiation and use is a critical consideration [60].

So far, the FDA has issued ruling on over 96% of the PMTAs submitted on or before the September 9, 2020 deadline [60], including 295 marketing denial orders (MDOs) for more than 1,089,000 flavored ENDS products [62]. Products subject to a MDO for a premarket application may not be introduced or delivered for introduction into interstate commerce. If the product is already on the market, the product must be removed from the market or risk enforcement [63].

On October 12, 2021, the FDA granted permission to R.J. Reynolds (RJR) to market its Vuse Solo closed ENDS device and two accompanying tobacco-flavored e-liquid pods with a nicotine strength of 4.8%, which approximates the nicotine content of a pack of cigarettes [63]. The FDA simultaneously issued MDOs for ten other flavors submitted under Vuse Solo but declined to state which ones [63]. RJR is the second-largest tobacco company in the United States (behind Altria), and a wholly owned subsidiary of Reynolds American Inc., after merging with the U.S. operations of British American Tobacco in 2004 (https://rjrt.com/). In a statement announcing the decision, the FDA said that “The authorized products' aerosols are significantly less toxic than combusted cigarettes based on available data.” The statement concluded that “For these products, the FDA determined that the potential benefit to smokers who switch completely or significantly reduce their cigarette use, would outweigh the risk to youth, provided the applicant follows post-marketing requirements aimed at reducing youth exposure and access to the products” [63].

The FDA's decision came on the heels of the 2021 NYTS report showing an estimated 2.06 million U.S. middle and high school students as current users of e-cigs, with Puff Bar, Vuse, and Juul among the most popular brands [19]. In its decision, the FDA stated that it was aware that 10% of high school students who used e-cigs, named Vuse as their favorite brand in the 2021 NYTS [63]. Vuse has become the fastest-growing e-cig brand, accounting for over 26% market share in the top five markets, being the market leader in Canada, the UK, France, and Germany [64]. In the United States, Vuse continues to outperform other brands, becoming the second largest (after Juul) and fastest-growing player in the market in 2021. Currently, Vuse owns 33% of the market share in the United States whereas Juul owns 40% [65].

The FDA's decision to grant marketing orders for Vuse Solo and accompanying e-liquid pods marks a milestone in vaping regulations as this is the first time ever that a set of vaping products gets clearance from the agency. The cleared products can now be mass marketed and sold legally in the United States. As expected, the watershed decision by the FDA to greenlight Vuse Solo vaping products has been praised by the industry as well as proponents of e-cigs for harm reduction. Simultaneously, there have been swift and harsh criticisms of the decision by many experts in public health and medicine and numerous healthcare advocacy groups [66].

As the FDA continues its premarket reviewing of the remaining PMTAs for ENDS products, including major brands such as Juul, and while teens' favorite products, specifically Puff Bars (i.e., disposable e-cigs in fruity and candy flavors, such as the highly popular “Blueberry Ice”), are being ordered off the market [67], Vuse is expected to continue its growth momentum owing to its “first-mover” advantage and industry-leading FEELM ceramic coil.

## Concluding Remarks and Recommendations

A growing body of research shows the disease-causing potential of vaping [24, 30–34, 34, 37]. However, new data are also emerging indicating that provision of free e-cigs in the context of randomized controlled trials is significantly associated with increased smoking cessation [29, 46, 68, 69]. Future research should weigh the health risks of vaping against its benefits based on three equally important factors: [1] how vaping affects youth initiation of cigarette smoking, nicotine-dependence, or other substance use (e.g., marijuana use); [2] efficacy of vaping in adult cessation of cigarette smoking; and [3] overall toxicity of e-cigs as compared to tobacco cigarettes in chronic users as well as the effects of “*exclusive”* vaping or smoking vs. dual use (i.e., co-use of e-cigs and combustible cigarettes). The net effect of vaping on the public's health can be determined when all the three factors are thoroughly investigated, both individually and in relation to one another, but not simply by examining each factor in a vacuum. The goal should be to prevent youth vaping while improving smoking cessation strategies. In smokers who are unable or unwilling to quit smoking, the objective should be to provide “*provably”* safe or less-harmful alternatives, which should “completely” or “substantially” substitute tobacco cigarettes.

## Data Availability Statement

The original contributions presented in the study are included in the article, further inquiries can be directed to the corresponding author/s.

## Author Contributions

AB conceived the study, performed literature search, and wrote the manuscript.

## Funding

This work was supported by grants from the National Cancer Institute of the National Institutes of Health (1R21CA268197) and the University of California Tobacco-Related Disease Research Program (28IR-0058 and T31IR-1839) to AB. The sponsors of the study had no role in study design, data collection, data analysis, data interpretation, writing of the report, or in the decision to submit for publication.

## Conflict of Interest

The author declares that the research was conducted in the absence of any commercial or financial relationships that could be construed as a potential conflict of interest.

## Publisher's Note

All claims expressed in this article are solely those of the authors and do not necessarily represent those of their affiliated organizations, or those of the publisher, the editors and the reviewers. Any product that may be evaluated in this article, or claim that may be made by its manufacturer, is not guaranteed or endorsed by the publisher.
